# Epigenetic Impact on EBV Associated B-Cell Lymphomagenesis

**DOI:** 10.3390/biom6040046

**Published:** 2016-11-24

**Authors:** Shatadru Ghosh Roy, Erle S. Robertson, Abhik Saha

**Affiliations:** 1 Department of Life Sciences, Presidency University, 86/1, College Street, Kolkata 700073, West Bengal, India; shatadru54@gmail.com; 2 Department of Otorhinolaryngology-Head and Neck Surgery, and the Tumor Virology Program, Abramson Comprehensive Cancer Center, Perelman School of Medicine at the University of Pennsylvania, Philadelphia, PA 19104, USA

**Keywords:** Epstein-Barr virus, B-cell lymphoma, Epigenetics

## Abstract

Epigenetic modifications leading to either transcriptional repression or activation, play an indispensable role in the development of human cancers. Epidemiological study revealed that approximately 20% of all human cancers are associated with tumor viruses. Epstein-Barr virus (EBV), the first human tumor virus, demonstrates frequent epigenetic alterations on both viral and host genomes in associated cancers—both of epithelial and lymphoid origin. The cell type-dependent different EBV latent gene expression patterns appear to be determined by the cellular epigenetic machinery and similarly viral oncoproteins recruit epigenetic regulators in order to deregulate the cellular gene expression profile resulting in several human cancers. This review elucidates the epigenetic consequences of EBV–host interactions during development of multiple EBV-induced B-cell lymphomas, which may lead to the discovery of novel therapeutic interventions against EBV-associated B-cell lymphomas by alteration of reversible patho-epigenetic markings.

## 1. Introduction

Epigenetics is defined as somatically-heritable changes causing alteration in gene expression patterns that are not exclusively reliant on simple gene sequence (recently reviewed in [1]). These alterations triggered by different external stimuli—ranging from multiple genotoxic stresses to infections caused by several intracellular pathogens, including tumor viruses—are stable and cell type-specific but may not always be heritable. Following the initial observation of DNA methylation as one of the most critical aspects for the stable maintenance of gene expression pattern, today epigenetic regulation has earned a notable place in scientific research on cellular and physiological response mechanism towards multiple external stimuli, particularly in disease biology. A number of cellular epigenetic mechanisms have shown to be determining factors for phenotypic variations—such as covalent histone modifications, DNA methylation, and genetic alteration by non-coding RNAs. DNA hypermethylation at the C-5 position of cytosines, deacetylation of histone tails, methylation of certain lysine or arginine residues of core histones and interaction with Polycomb repressor complexes (PRCs) play important roles in promoter silencing or formation of the ‘heterochromatin structure’. In contrast, transcriptionally-active promoters are represented by CpG-hypomethylation along with specific euchromatic histone modifications such as acetylation and methylation of core histones—H3 and H4 at the active site residues [1,2,3]. An indirect DNA hypomethylation pattern can also be established by inhibition of DNA methyltransferase (DNMT) activities—particularly targeting the DNMT enzyme, DNMT1. Histone acetylation is reversed by histone deacetylase (HDAC) activities [1,2,4].

One of the hallmarks of cancer development is incompetency at the cell-cycle checkpoints during cell division that controls the overall ‘cell numbers’ in response to the needs of the organism. Inapposite functions of genes that promote (proto-oncogenes) or inhibit (tumor-suppressors) cell growth can be initiated by either genetic or epigenetic deregulations. Different types of cancer engage diverse epigenetic mechanisms, ranging from an altered methylation profile at the CpG island, to histone modifications, to deregulation of DNA binding proteins in order to attenuate transcription of tumor suppressor genes while activating oncogenes [5].

It is now known that seven human viruses, Epstein–Barr virus (EBV), human papilloma virus (HPV), hepatitis B virus (HBV), human T-cell lymphotropic virus (HTLV-1), hepatitis C virus (HCV), Kaposi’s associated sarcoma virus (KSHV), and Merkel cell polymavirus (MCPyV) contribute to approximately 20% of the cancers worldwide. EBV (or human herpes virus 4; HHV4), a member of γ-*Herpesviridae* subfamily, is associated with a number of human malignancies [6]. It was first detected in 1964 by Michael Anthony Epstein and Yvonne Barr in cells derived from Burkitt’s lymphoma (BL), a B-cell childhood malignancy that is endemic in Equatorial Africa. Later several epidemiological studies have demonstrated that more than 95% of the global population is infected with this virus with no apparent symptoms. However, in immunocompromised individuals—such as post-transplant and acquired immune deficiency syndrome (AIDS) patients—EBV can cause a wide spectrum of human cancers, both of hematopoietic and epithelial origin. Although EBV can infect various cell types, such as T-lymphocytes and endothelial cells [7,8,9,10], EBV preferably infects B-lymphocytes and is responsible for a wide spectrum of B-cell lymphomas, including Hodgkin’s lymphoma (HL), Burkitt’s lymphoma (BL), post-transplant lymphoproliferative disorders (PTLDs), diffuse large B-cell lymphomas (DLBCLs), and various AIDS-associated B-cell lymphomagenesis (Table 1 and reviewed in [11]). In vitro EBV infection in resting B-lymphocytes (RBLs) can result in cellular immortalization and subsequent transformation, known as lymphoblastoid cell lines (LCLs). LCLs are being used as model system for studying the role of EBV encoded latent antigens in B-cell transformation and subsequent B-cell lymphoma development [11]. Similar to the other human cancers, epigenetic modifications were shown to play a critical role in EBV-associated B-cell lymphoma development [12,13]. An in depth analysis of these epigenetic alterations would certainly enhance our present understanding of viral pathogenesis and provide clues for future therapeutic strategies against a wide spectrum of EBV associated B-cell lymphomas as described in Table 1.

## 2. Epigenetic Regulation during EBV Lytic Replication

During initial infection, the lytic replication cycle is triggered by the expression of two major viral immediate early genes—BRLF1 (encodes Rta) and BZLF1 (encoded Zta)—which further activate transcription of several viral early and late genes required for viral DNA replication and structural building blocks [18]. Moreover, in latently-infected cells, intermittent expression of these genes also plays a critical role in establishing viral pathogenesis by infecting surrounding newer cells [18]. A number of positive and negative transcriptional control elements influence BZLF1 expression. BZLF1, a member of bZIP family of transcription factors, binds to the lytic origin of viral replication—oriLyt along with specific sites present in BZLF1 and BRLF1 promoters (Zp and Rp, respectively). Collectively, BZLF1 and BRLF1 transcriptionally activate a series of early EBV promoters and play a central role in viral lytic replication [19,20,21]. Therefore, it is conceivable that silencing of these early gene promoters would be necessary in establishing the latency programs [20]. As expected, in EBV-associated neoplasms, the immediate early promoters are shown to be typically hypermethylated along with deposition of other promoter repressive elements. For example, EZH2, a member of PRC2 repressive complex, is recruited to the BZLF1 promoter during latency to subsequently facilitate the tri-methylation of lysine 27 on histone H3 (H3K27me3) associated transcription silencing [22]. Additionally, several HDAC inhibitors were shown to induce viral lytic replication from both naturally established EBV positive BL lines, as well as in vitro-generated LCLs, indicating the role of histone acetylation in regulating BZLF1 promoter [22]. Unlike other transcription factors, an interesting feature of BZLF1 is that it generally binds to the methylated promoter sequence and initiates temporal viral replication in latently-infected cells. Upon successful reactivation, BRLF1, which prefers rather completely un-methylated DNA, starts transactivation and DNA replication [21].

## 3. Epigenetic Regulation during EBV Latency

During latency, only a limited number of EBV promoters are active in a specific cell type, resulting in different gene expression patterns—known as their ‘latency program’. These programs can be categorized into several groups—0, I, II, and III—depending upon the cell-type. The latent promoters are strictly regulated by the cellular epigenetic machinery and demonstrate characteristic gene expression patterns (reviewed in [11,23]). Reversible epigenetic silencing of EBV-encoded latent genes thus exert a survival strategy from immune responses in cooperation with controlling B-cell maturation, ultimately leading to B-cell lymphoma development. In vitro EBV-transformed LCLs resembling B-cell lymphomas in particularly immunosuppressive individuals, express a latency III program (also referred to as ‘growth program’), which includes a full complement of latent genes encoding six nuclear proteins (Epstein–Barr nuclear antigen (EBNA)-1, -2, -3A, -3B, -3C, and -LP), three integral latent membrane proteins (LMP-1, -2A, and -2B), along with approximately thirty microRNAs (miRNAs) from two different clusters (BHRF and BamHI-A region rightward transcript (BART)) and two non-coding RNAs (EBV-encoded small RNA (EBER)-1 and -2). In addition to LCLs, the type III latency program is typically detected in EBV-associated PTLDs and AIDS-associated lymphomas. The type II latency program, with further restricted viral gene expression patterns of EBNA-1, LMP-1, and LMP-2, is predominantly found in HL, nasopharyngeal carcinoma (NPC), and gastric carcinoma (GC). The type I latency program primarily expresses EBNA-1 and is associated with only BL. The type 0 latency program is restricted only in RBLs of germinal center and associated with variable expression of EBNA-1 and, perhaps, also LMP-2A. During the B-cell differentiation process latency programs can change from latency III to latency 0 in the long-living memory B-cells, where EBV episomes can persist for the life of the infected host [11,23].

### 3.1. Epigenetic Regulation of EBV Latency Promoters

DNA methylation is largely involved in regulating the EBV latent promoters [24]. Wp (located at the 5′ end of BamHI W repeats) represents the first viral promoter to be activated and initiates the transcription of EBNA-2. Subsequently, Wp-initiated transcripts are slowly decreased by CpG methylation and Cp takes over as the predominant EBNA promoter expressing transcripts for all six EBNAs which, in turn, regulates transcription from LMP promoters [24]. EBNA-2 plays an essential role in this promoter switching [25]. In addition to Cp, EBNA-2 also transactivates the promoters of LMP-1 and LMP-2 through association with cellular transcription factors, including recombining binding protein suppressor of hairless (RBP-Jk), PU.1, and several HAT components—p300, CBP, and p300/CBP-associated factor (PCAF). The LMP promoters are also additionally controlled by CpG hypermethylation, as observed in various cell types. Although not completely understood, a number of studies have suggested both type I and type II latency programs arise through epigenetic silencing of the Cp and LMP-1 promoter by DNA methylation and histone deacetylation. For instance, in type III latency program the active state of these promoters are designated as preoccupied for H3K9Ac, H3K27ac, and H3K4me3, and these euchromatic marks are absent in other latencies where these promoters are inactive [25].

While in type III latency, EBNA-1 transcripts are generated from Cp promoter, and in type I latency EBNA-1 expression is largely controlled by Qp [15]. Interestingly, unlike other latent promoters, DNA-methylation does not play a role in deactivation of Qp, a promoter which, so far, has been shown to only drive EBNA-1 expression. Since EBNA-1 is required for viral replication and episome maintenance in latently-infected cells, its expression appears to be associated with all major latency programs. In addition, EBNA-1 also transactivates EBNA-2 and LMP-1 [15]. EBNA-1 can coordinate the switch between the latency programs through recruitment of cellular factors. Using a genome wide chromatin immunoprecipitation (ChIP) assay, chromatin insulator protein, CTCF was found to bind immediately upstream of the EBNA-1 binding sites in Qp, implicating an important role for CTCF in preventing epigenetic silencing of Qp in B-cells [15]. In addition, CTCF was later shown to be involved in controlling LMP-1 and LMP-2 promoter selection through epigenetic regulation during latency [26].

The euchromatic marks frequently associated with active Cp, Qp, and LMP promoters are acetylation of histones H3 and H4 and methylation pattern of H3K4me2 or H3K4me3. The EBER genes closely upstream of oriP are also constitutively unmethylated and expressed in all latency programs [15]. The immediate-early promoter Zp required for initiation of lytic cycle is also found to be associated with specific heterochromatic histone mark (H3K27me3 and H4K20me3) during latent infection in B-cells [22].

### 3.2. Epigenetic Regulation by the Viral Oncoproteins

**Figure 1:**
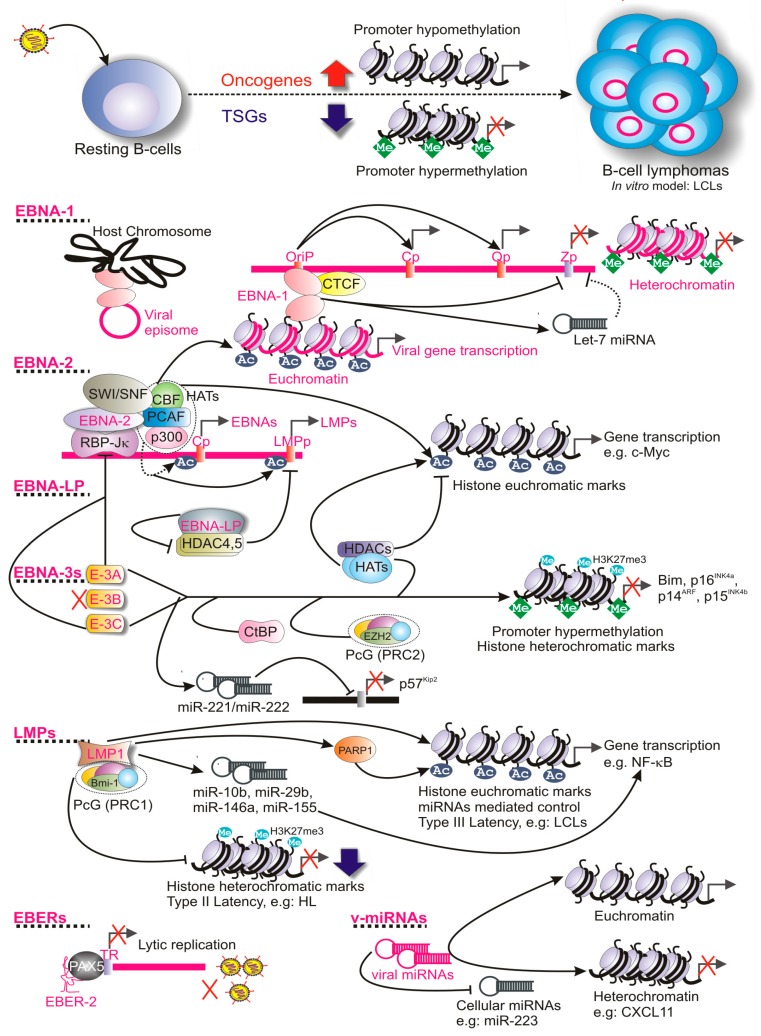
Epstein-Barr virus (EBV) latent transcripts mediated epigenetic regulation during B-cell lymphomagenesis. EBV infection in resting B-cells (RBLs) led to a general up-regulation of oncogenes and down-regulation of tumor suppressor genes (TSGs). EBV nuclear antigens , including EBNA-1, -2, -3A, -3C, -LP, and Latent membrane protein 1 (LMP-1) extensively modulate both viral and cellular gene transcription through epigenetic regulation, leading to B-cell transformation and subsequent B-cell lymphoma development. EBV non-coding RNA, EBER-2, through interaction with B-cell transcription factor PAX5, bind at the viral terminal repeat (TR) region and thereby blocks lytic cycle activation. EBV encoded several microRNAs (miRNAs) that are also involved in gene transcription and thereby contributing to the B-cell lymphomagenesis. Viral DNA and genes/proteins are indicated as pink and cellular DNA and genes/proteins are indicated as black. LCLs: Lymphoblastoid Cell lines; OriP: Origin of replication P; CXCL11: C-X-C motif chemokine ligand 11.

Reverse genetic studies indicate that five viral oncoproteins including EBNA-1, -2, -3A, -3C, and LMP-1, among all latent transcripts, are absolutely essential for in vitro B-cell transformation and LCL growth maintenance (reviewed in [11,23]). Herein, we review on how viral latent transcripts employ multiple epigenetic mechanisms in regulating different latency programs and associated B-cell lymphomagenesis. Some of the important functions are tabulated in Table 2 and portrayed in Figure 1.

#### 3.2.1. Role of EBNAs

EBNA-1 is the only latent protein expressed in all EBV-associated tumors and latency programs [15]. As described earlier, EBNA-1 acts as a potent epigenetic regulator controlling viral transcription from both Cp and Qp. In addition to regulating viral transcription, EBNA-1 initiates viral replication from oriP, which lacks CpG methylation and serves as long distance enhancer region for subsequent promoter selection between Cp and Qp [15]. The transactivation property of EBNA-1 can be correlated to its interaction with bromodomain-containing protein 4 (Brd4), ,which preferentially interacts with acetylated chromatin [41]. EBNA-1 also recruits a histone-deubiquitination complex to oriP for regulating viral replication and episome maintenance [42]. Moreover, by inducing let-7 miRNA and repressing Dicer expressions, EBNA-1 was shown to block lytic cycle gene activation [43]. Recently, a genome wide ChIP-Seq and RNA-Seq data revealed that EBNA-1 binds nearby 1000 different sites on human chromosome [15]. As similar to the regulation on the viral episome, EBNA-1 also alters host chromatin structure and nucleosome positioning and induces a more open chromatin structure preferable for host gene transactivation, implying a direct role in EBV-induced pathogenesis [15].

EBNA-2 functions as one of the major EBV-encoded transcriptional activators through recruiting sequence specific transcription factors (TFs), including RBP-Jκ and PU.1, and HATs, like p300, CBP and PCAF, to modulate both viral and host gene expression [44,45]. Moreover, EBNA-2 recruits SWItch/Sucrose Non Fermentable (SWI/SNF), a nucleosome remodeling factor, to open up chromatin conformation and thereby inducing gene transcription, such as cMyc [27,46]. Although proved to be non-essential during in vitro B-cell transformation, the importance of EBNA-LP can be explained in the fact that it is the first transcript during the course of latency establishment, which in turn acts as a co-transactivator of EBNA-2 expression by deregulating HDAC (HDAC4 and HDAC5) activities [25,31]. EBNA-3, comprising of three tandem repeat genes namely -3A, -3B, and -3C, was generated by alterative splicing events of a long transcript initiated from the Cp promoter during the type III latency program associated with various B-cell lymphomas, particularly in immune-compromised situations [13].

Although EBNA-3 proteins share little significant sequence homology and redundant functional consequences, only -3A and -3C influence B-cell lymphomagenesis through extensive chromatin modification and recruitment of several critical B-cell TFs, such as RBP-Jκ [27,47,48,49,50,51]. The potential involvement of EBNA-3 proteins in epigenetic reprogramming was first established by its interaction with several HATs and HDACs including p300, CBP and HDAC1/2 proteins [29,52,53]. Genome-wide micro-array profile and ChIP-Seq data revealed that both EBNA-3A and -3C epigenetically regulate over 1000 cellular genes, of which most are transcriptionally down-regulated [27,54,55]. In addition, a number of independent studies have demonstrated that these EBNA-3 proteins transcriptionally repress several tumor suppressor genes (TSGs) through promoter hypermethylation, histone modification—particularly H3K27me3 and recruitment of PRC [12,56]. EBNA-3A and -3C deregulated TSGs, including several cyclin dependent kinase inhibitors (CDKIs) p14^ARF^, p15^INK4a^, and p16^INK4a^ (reviewed in [13,57]). Importantly, while transcriptional repression of p16^INK4a^ plays a central role during EBV mediated B-cell transformation at least in an in vitro system; exact roles for p15^INK4b^ and p14^ARF^ suppression remain yet to be evaluated in more detail [58,59]. Additionally, for proper deposition of the H3K27me3 repressive epigenetic mark on the p16^INK4a^ gene locus, C-terminal-binding protein 1 (CtBP1) recruitment by EBNA-3 proteins plays a crucial role [59]. In a separate study, EBNA-3A was shown to recruit MIZ1 and H3K27me3 histone modification for inhibiting p15^INK4b^ gene transcription [60]. In addition to p16^INK4a^, another well-studied example of EBNA-3-mediated epigenetic suppression is Bim/BCL2L11 tumor suppressor expression [28,30]. Using genetically modified EBV particles lacking EBNA-3 expressions, it was demonstrated that both EBNA-3A and -3C are responsible for transcriptional repression from the BCL2L11 locus through facilitating CpG methylation, recruitment of PRC2 core subunits, and deposition of H3K27me3 heterochromatic marks at the promoter region [12,28]. Although there is no direct interaction, recently EBNA-3A and -3C were shown to recruit PRC2 methyltransferase EZH2 activity for Bim transcriptional repression [12,27]. Moreover, EBNA-3A and -3C were shown to induce miRNA cluster miR-221/miR-222 to down-regulate p57^Kip2^ expression in B-cells [61].

#### 3.2.2. Role of LMPs

While LMP-1 mimics Cluster of differentiation 40 (CD40) signaling, LMP-2A mimics B-cell receptor signaling pathways in latently-infected B-cells [62,63]. LMP-1 through transactivation of the nuclear factor-κB (NF-κB)-mediated signaling pathway regulates an overall gene expression pattern in HL and LCLs [64]. One of the LMP-1-induced genes is the PRC1 component Bmi-1, which contributes to the survival of HL cells through its influence on transcriptional regulation of several other genes [65]. LMP-1 was also shown to induce a histone demethylase, lysine demethylase 6B (KDM6B) which, in turn, reduces H3K27me3 heretochromatic marks in type I latency expressing HL cells [66]. Very recently, LMP-1 was shown to regulate cellular gene expressions by reducing histone heterochromatic mark H3K27me3 through poly (ADP-ribose) polymerase 1 (PARP1) activation in type III latency [32]. Additionally, LMP-1 affects gene transcription and subsequent B-cell lymphomagenesis through activation of multiple miRNAs, including miR-10b, miR-29b, miR-146a, and miR-155 [33]. While both LMP-1 and -2A were shown to elevate DNMT-1 expression in NPC lines with the latency II program, at least one viral antigen, LMP-1, was further shown to down-regulate DNMT-1 expression in EBV infected germinal B-cells [67,68].

#### 3.2.3. Role of EBERs

EBV expresses two small abundant nuclear non-coding RNAs—EBER-1 and EBER-2 [69]. While roughly the entire EBV episome was shown to be largely methylated, the EBER regions are somehow devoid of this modification and, as a result, expressed in all forms of latency programs, as well as in the lytic cycle [69]. Although genetically-engineered EBV particles lacking EBER expressions provide conflicting observations, it has been suggested that EBERs, particularly EBER-1, through associating with a number of cellular factors, play an important role in B-cell lymphomagenesis. For example, while both EBERs form complexes with host RNA chaperone protein La, EBER-1 was shown to specifically interact with ribosomal protein L22 and AU-rich element RNA-binding protein 1/heterogeneous nuclear ribonucleoprotein D0 (AUF1/hnRNPD) [35,36,37]. Nevertheless, the precise molecular mechanism by which EBERs exert pathogenesis has not been fully understood. Recently, a genome wide screen using CHART (Capture Hybridization Analysis of RNA Targets) technology, demonstrated that EBER-2, through interacting with PAX5, was specifically localized at the terminal repeat (TR) region of EBV episome, thereby affecting both viral gene transcription and lytic replication with possible implications in oncogenesis [38]. Since, PAX5, a cellular TF, has been portrayed as a critical regulator of B-cell differentiation and development [70], one could argue regarding the importance of EBER-2 in EBV associated B-cell lymphomagenesis.

#### 3.2.4. Role of Viral miRNAs

Recent studies have expanded our understanding of EBV miRNAs. EBV was the first virus in which miRNA was detected [71]. So far, two independent EBV-miRNAs are known—the miR-BHRF1 family consisting of four miRNAs, and the miR-BART family located in the introns of the BART [72,73]. While BART miRNAs are primarily expressed in BL with the type I latency program, and in NPC with the type II latency program, BHRF1 miRNAs are typically expressed in B-cell lymphomas undergoing the type III latency program (reviewed in [23,74]). However, in a recent study miR-BARTs were shown to be largely expressed in immunodeficiency-associated Burkitt’s lymphomas (ID-BLs) with the type I latency program [75]. Overall, EBV-encoded miRNAs play important roles in regulating gene expression, evading the host immune system, inhibiting apoptosis, and metastasis development [74]. For example, during the early phase of B-cell infection, BHRF1 miRNAs block apoptosis and induce cell-proliferation [76]. In primary B-cell lymphomas, miR-BHRF1-3 may also contribute to the down-regulation of the T-cell attracting chemokine CXCL11 [39]. In NPC lines, miR-BART5 inhibits apoptosis by transcriptional repression of several pro-apoptotic genes—including p53 upregulated modulator of apoptosis (PUMA) and BCL2L11 (BIM) [40]. Recently, miR-BART6-3p was shown to influence the global gene expression profile of EBV-positive BLs and, further, it was shown to induce apoptosis [77,78]. Although viral miRNAs generally do not share any sequence homology with cellular miRNAs, there are examples of a few viral orthologs to human miRNAs. In an in silico analyses, miR-BART-5 was found to be an ortholog of cellular miR-18 [79]. In spite of their low sequence homology, a number of viral miRNAs were shown to compete with cellular miRNAs for transcriptional regulation. For example, miR-BART15 targets miR-223 binding sites of the NLRP3 inflammasome at its 3′-untranslated region (UTR), implying a potential role in inflammation [80].

## 4. Epigenetic Profiles of Cellular Genomes during EBV-Induced B-Cell Lymphomagenesis

### 4.1. Early Infection

DNA-hypermethylation at the CpG islands of TSGs represents one of the attractive mechanisms of cancer progression (reviewed in [81]). Likewise, several TSGs’ hypermethylation are also demonstrated in EBV-associated cancers including multiple B-cell lymphomas [28]. Recently we have demonstrated that EBV infection of RBLs resulted in overall transcriptional repression of a panel of TSGs through recruiting hypermethylation activity [82]. Among 96 gene promoter sequences assessed, approximately 50% of the TSGs were shown to have an increasing trend of methylation in response to EBV infection. The list includes ABCB1, AKT1, ATM, BCL2, BRAF, CADM1, CDKN1A, CDKN1B, CDKN2A, CDKN2B, CTNNB1, DAPK1, DIRAS3, EGFR, ERBB2, HIC1, HRAS, ING1, MDM2, MGMT, MLH1, MYCN, NF1, NF2, NME1, OPCML, PRDM2, PTCH1, RB1, RET, RUNX3, SCGB3A1, SFRP2, SH3PXD2A, SMARCB1, TGFB1, TGFBR2, THBS1, TP53 and TP73. While only six TSGs—APC, HOXA1, MYC, SFRP1, SOCS1, and WT1—demonstrated a decreasing trend in methylation, 25 TSGs, including ABL1, CAV1, CCND2, CDH13, CDX2, DKK3, DLC1, E2F1, GSTP1, IGF2, IGF2R, NFKB1, PTEN, PTGS2, RASSF1, SFN, SLC5A8, MEN1, TERT, TIMP3, TSC1, TSC2, VHL, WWOX, and ZMYND10, fall in rather an unusual category where methylation status was either significantly elevated by two days post-infection followed by down-regulation, or in reverse [82]. The importance of second-day post-infection was elaborated as EBV hyper-proliferation [83], which might explain this fluctuation during the course of EBV infection in RBLs. Additionally, EBV infection in RBLs demonstrated a global alteration of transcriptional expression of chromatin remodeling enzymes and other accessory cellular proteins [82]. Importantly, EBV infection of RBLs leads to an elevated expression of DNMT-3A, -3B along with -3L, but not DNMT-1 [82]. DNMT-3L, however, does not directly participate in CpG methylation due to the absence of its catalytic domain, and forms stable complexes with both DNMT-3A and -3B in order to increase their activities [4]. DNMT3A, however, was expressed at a lower level as compared to DNMT3B and DNMT3L, suggesting that EBV infection of RBLs may engage a DNMT-3B/-3L mediated transcriptional repression complex. While DNMT-1 binds to hemimethylated DNA and maintains the methylation status, DNMT-3A and DNMT-3B function as de novo methyltransferases [4]. Elevated expression of one or more of the DNMTs has been clearly demonstrated in many cancers [4]. Interestingly, the transcript levels of CpG-dinucleotide methyl binding domain (MBD) genes MBD1, MBD3, MBD4, and MeCP2, but MBD2 were significantly depleted. In addition, the HDACs—a subset of 11 transcripts—were, in general, markedly up-regulated by two days post-infection and maintained the level throughout the course of infection [82]. Overall, these results provide additional clues that chromatin modifying and remodeling factors may function in collaboration with methylation activities to regulate TSGs expression during EBV infection of RBLs. In vitro EBV transformed LCLs treated with demethylating agents—DNMT inhibitor 5′-azacytidine—can revive the transcription of these TSGs, implying for future therapeutic interventions [82].

### 4.2. Lymphoblastoid Cell Lines

As earlier discussed, LCLs provide a suitable in vitro model for exploring EBV-induced B-cell lymphomagenesis particularly seen in those originated from immunocompromised patients suffering from PTLD or several AIDS-associated lymphomas [11,49,50,57].

#### 4.2.1. Host miRNAs

There is increasing evidence of a role for miRNAs in B-cell differentiation and B-cell lymphoma development, including those which are EBV associated. EBV infection in RBLs leads to global alteration of host miRNA expressions and contribute to the overall lymphomagenesis. A number of studies have previously addressed the role of EBV infection on host miRNAs expressions. In a recent study with a qPCR-based panel containing over 375 miRNAs, a significant differential expression pattern was observed among 34 host miRNAs. Of these, while 24 miRNAs including miR150, miR199a-5p, miR-223, miR-28-5p, miR-451, were significantly depleted, 10 miRNAs including miR-34a, miR-155, miR-193b, miR-365, miR-551b, displayed induction. The induction and repression of miRNAs are shown to be regulated by the NF-κB pathway through alteration of histone-methylation—particularly H3K4me3 and H3K27me3. Interestingly, a similar miRNAs expression profile was observed when RBLs were either induced by interleukin 4(IL4)/CD40 ligand (CD40L) or infected with genetically-engineered EBV lacking EBNA-2 or LMP-1 open reading frames (ORFs). Since, LMP-1 was best known for induction of the NF-κB cannonical pathway, it has been suggested that stimulation of the NF-κB pathway could take place by mutant LMP-2A EBV infection. Earlier, miR-155 expression was shown to be drastically elevated in EBV transformed LCLs and DLBCLs in comparison to EBV-positive BL tumors latency I program, indicating that miR-155 plays an important role in EBV-associated B-cell lymphomas in an immunocompromised environment. In addition, knockdown of miR-155 expression in LCLs resulted in cell-cycle arrest and subsequent apoptotic induction, possibly through targeting BMP-signaling pathways. Among other deregulated miRNAs known to target B-cell differentiation, miR-34a transactivates forkhead box P1 (Foxp1), whereas miR-150 expression is negatively regulated by c-Myb expression. Another group showed that miR-146a is up-regulated by LMP-1 through a canonical NF-κB signaling cascade. Overall, these studies indicate a strong correlation of EBV latent oncoproteins with miRNA-mediated acquisition of the phenotype in B-cells and subsequent lymphoma development.

#### 4.2.2. Super-Enhancers

Super-enhancers are clusters of gene-regulatory sites characterized by several activation TFs, H3K27ac modification, and recruitment of bromodomain binding protein, BRD4, or mediator of RNA polymerase II transcription subunit 1 (Med1), that regulate several cellular signaling, including gene transcription, differentiation, and cancer development. Earlier, in several lymphoid cancers, such as multiple myeloma and DLBCLs, the *MYC* oncogene was shown to be regulated by super-enhancers. Very recently, using ChIP-Seq EBV-specific super-enhancers were identified in LCLs [55]. Four EBV-essential oncoproteins (EBNA-2, -LP, -3A and -3B) and five previously demonstrated EBV LMP-induced NF-κB subunits (RelA, RelB, cRel, p50, and p52) co-occupied ~187 enhancer sequences and are referred to as ‘EBV super-enhancers’ based on H3K27ac signal intensity. The physiological significance of these super-enhancers was evaluated by attenuating functions of either viral oncoprotein EBNA-2 or NF-κB [55]. While EBNA-2 was shown to be specifically involved in MYC expression, NF-κB positively regulates BCL2 expression [27,55]. Overall, these findings provide further insights in our understanding of EBV-induced B-cell transformation and subsequent lymphoma development.

### 4.3. Burkitt’s Lymphoma (BL)

BL is an aggressive childhood lymphoid malignancy that can be classified into three clinical forms, such as endemic (eBL), sporadic (sBL), and immunodeficiency associated (ID-BL). In addition to the characteristic feature of reciprocal translocation of *MYC* in one of the three immunoglobulin (Ig)-genes, EBV is believed to play an important role in BL pathogenesis [84]. Unlike other EBV associated B-cell malignancies, BL typically expresses EBNA-1 along with several miRNAs within the BART family. The epigenetic profile of BL tumors appears to significantly deviate from that of LCLs, indicating genetic predisposition might play a role in determining epigenetic marks. A comprehensive methylation profile of several TSGs was established in hundreds of BL tumor patient’s biopsy samples [85]. In contrast, until now, only one completed ‘bisulfite sequence’ profile was established in an EBV-positive endemic BL line (Daudi), in which nearly 69% of total genomic CpG-islands were shown to be methylated [86]. However, whether primary BL tumors exhibit the same epigenetic profile as Daudi remains unexplored. In an attempt to determine overall genetic mutations that contribute to BL progression, around 59 BL, along with 94 DLBCL patient’s genome samples, were sequenced [87]. Among 70 susceptible genes, mutations found in chromatin remodeling SWI/SNF family members—*ARID1A* and *SMARCA4*—were found to be associated with roughly 25% of BL-tumors [87], suggesting genetic alterations govern further epigenetic deregulations during BL pathogenesis. In another recent study by Navari et al. using several ID-BL patient samples including both EBV-positive and -negative, around 252 genes were differentially expressed [75]. In addition, besides expression of 18 EBV-BART transcripts, 10 cellular miRNAs (miR-16, miR-26a, miR-142-5p, miR-148a, miR-200b, miR-223, miR-668, miR-877, miR-1178, and miR-1233) were shown to have an elevated expression pattern in EBV positive ID-BL [75]. Overall, it appears that both viral coding (EBNA-1) and non-coding (miRNAs) transcripts, together with HIV-co-infection, play a central role in ID-BL pathogenesis [75].

### 4.4. Hodgkin’s Lymphoma (HL)

Both genetic and epigenetic predispositions are shown to be necessary for EBV-induced HL development. Studies suggested that in vitro EBV-infected germinal B-cells can serve as a model system for investigating EBV-associated HL pathogenesis [68]. Leonard et al. showed that DNMT-3A, but not DNMT-3B nor DNMT-1, was elevated both in EBV-infected germinal B-cells, as well as in HL cell lines [68]. Using transient transfection assay, LMP-1 was shown to be responsible for DNMT-1 down-regulation. However, the underlying mechanisms that govern this deregulated expression pattern of DNMT-3A and -3B in HL lines was not identified in EBV-infected germinal B-cells. As discussed in the earlier section, Wp represents the initial promoter for EBNA expression during EBV-induced B-cell transformation, which, in the course of infection, is silenced due to hypermethylation and switches to Cp as predominant latent promoter [15]. Using pyrosequencing and ChIP analyses, DNMT-3A, but not DNMT-1 nor DNMT-3B, was shown to bind Wp and thereby maintain the type III latency program [68]. These results may, thus, serve as frontline prognostic markers for EBV-positive HLs and other associated B-cell lymphomas with type III latency profile. Of note, recently we also showed a significant DNMT-1 depletion in response to EBV infection in RBLs whereas, on the contrary, DNMT-3B, and not DNMT-3A, was elevated during in vitro EBV infection, as well as in transformed B-cells [82]. Interestingly, DNMT-1 activation appears not to be involved in EBV-induced B-cell lymphomagenesis, but plays an important role in other viral-infected human neoplasms, such as tumors induced by HPV or HBV [88,89].

## 5. Future Perspective

In cancer treatment, the utmost challenge lies in the amalgamation of epidemiological facts with laboratory findings that ultimately translate to a clinical perspective. It has only recently become apparent that deregulation of epigenetic mechanisms are frequent events in several B-cell lymphoma developments, including those which are associated with oncogenic viruses, such as EBV. In light of recent findings, these epigenetic marks provide critical insights into EBV induced B-cell lymphomagenesis and offer the possibility of functioning as both potential biomarkers and molecular targets for therapeutic intervention. With the advent of several drugs that specifically targets the ‘epigenetic machinery’ we certainly expect a meticulous expansion of current chemotherapy regimens against multiple EBV-associated B-cell lymphomas given the tremendous epigenetic reprogramming established in EBV infected B-cells.

## Figures and Tables

**Table 1:** EBV-associated B-cell lymphomas and associated gene expression patterns.

B-Cell Lymphomas	Activated Promoters	Latency Programs	Latent Transcripts	Refs.
Burkitt’s lymphoma (BL)	Qp, Cp	I	EBNA-1, EBERs	[14,15]
Hodgkin’s lymphoma (HL)	Cp, LMP-1p	II	EBNA-1, LMP-1/2, EBERs	[16,17]
AIDS-associated B-cell lymphomas	Wp, Cp, LMP-1p/2Ap	III	All EBNAs, LMPs, EBERs and miRNAs	[15]
Post-transplant lymphoproliferative disorder (PTLD)	Wp, Cp, LMP-1p/2Ap	III	All EBNAs, LMPs, EBERs and miRNAs	[15]
Diffuse large B-cell lymphomas (DLBCLs)	Wp, Cp, LMP-1p/2Ap	III	All EBNAs, LMPs, EBERs and miRNAs	[15]

AIDS: acquired immune deficiency syndrome; EBV: Epstein–Barr virus; EBER: EBV-encoded small RNA; EBNA-1: Epstein–Barr nuclear antigen-1; LMP: latent membrane protein; miRNA: microRNA; Qp: *Bam*HI Q promoter; Cp: *Bam*HI C promoter; Wp: *Bam*HI W promoter.

**Table 2:** Role of EBV latent transcripts in epigenetic deregulation during B-cell lymphomagenesis.

Latent Transcripts	Proposed Functions	Refs.
EBNA-1	Hypomethylation, gene activationAlters overall viral episome chromatin structure and nucleosome positioning	[15]
EBNA-2	Promoter activation though histone acetylation	[27]
EBNA-3A	BCL2L11 promoter repression through CpG-methylation and recruiting PRC2 complex, H3K27me3 heterochromatic mark Represses CDKN2A through recruiting CtBP, depositing H3K27me3 Transcriptional regulation through interacting with several HATs and HDACs Inhibits CDKN2B transcriptions through induction of H3K27me3 heterochromatic mark	[27,28]
EBNA-3C	BCL2L11 promoter repression through CpG-methylation and recruiting PRC2 complex, H3K27me3 heterochromatic mark Transcriptional regulation through interacting with several HATs and HDACs Represses p16^INK4A^ through recruiting CtBP, depositing H3K27me3	[27,28,29,30]
EBNA-LP	Transactivates EBNA-2 through displacing HDAC4/5 activities	[31]
LMP-1	Down-regulates DNMT-1 in HL lines and in vitro EBV infected germinal B-cells Transactivates through recruiting KDM6B, which facilitates H3K27me3 demethylation Elevates Bmi-I of PRC1 repressive complex, thereby causing promoter silencing Transactivates through reducing H3K27me3 repressive mark by PARP1 activation Upregulates several miRNAs and affects gene transcription	[32,33]
LMP-2A	May target miR-155 for regulating gene transcription	[34]
EBERs	Interacts with multiple cellular factors, thereby affecting the gene transcription and episomal replication	[35,36,37,38]
miRNAs	miR-BART6-3p regulates gene transcription in BL miR-BART15 competes with cellular miR-223 binding site, thereby affecting inflammasome formation	[39,40]

BCL2L11: Human BCL2 like protein 11; PRC2: Polycomb-group repressive complex 2; H3K27me3: Histone H3 lysine 27 trimethylation; CDKN2A: Cyclin dependent kinase inhibitor 2A; CtBP: C-terminal binding protein; HATs: Histone acetyltransferases; HDACs: Histone deacetylases; CDKN2B: Cyclin dependent kinase inhibitor 2B; DNMT-1: DNA methyltransferase 1; HL: Hodgkin’s lymphoma; KDM6B: Lysine specific demethylase 6B; Bmi-I: B lymphoma Mo-MLV insertion region 1 homolog (mouse); PRC1: Polycomb-group repressive complex 1; PARP1: Poly (ADP-ribose) polymerase 1; miR-155: microRNA 155; miR-BART6-3p: MicroRNA EBV BamHI-A rightward transcript 6-3p; miR-BART15: MicroRNA EBV BamHI-A rightward transcript 15; miR-223: MicroRNA 223.
